# Case of Carotid Aneurism, in Which Galvanism Was Applied to the Blood in the Sac by Means of Acupuncture

**Published:** 1847-01

**Authors:** John Hamilton

**Affiliations:** Surgeon to the Richmond Hospital


*Case of Carotid Aneurism, in which Galvanism was applied to the Blood in the Sac by means of Acupuncture.* By JOHN HAMILTON, M. R. I. A., Surgeon to the Richmond Hospital.—The relation of the following case may prove serviceable to those who may try the galvano-puncture in cases of aneurism. In the first trials of a new remedy, every case should be faithfully narrated, the unsuccessful as well as the successful, that the causes of failure in the first may be clearly recognised and avoided.

James Holmes, aged 43, admitted into the Richmond Hospital, March 26, 1846. He had formerly served as a soldier in the East Indies, and was, at the end of eleven years, sent home on account of bad health. When admitted, he presented the appearance of a man whose constitution had been completely broken down (as, in fact, it was) by climate, drinking, and the effects of the syphilitic poison. He had two soft nodes, one on the sternum, the other over one of the ribs. There was strumous enlargement of the lymphatic glands on the left side of the neck, with two or three fistulous openings from which thin pus flowed. He had diarrhœa, cough, headache, and restless nights; but the most distressing symptom of all was nearly constant vomiting of a greenish-yellow fluid, and of almost every thing he took. His complexion was of a pale straw colour, and he was so weak that he could scarcely stand. He had formerly been twice in hospital under my care, once for a large abscess in the buttock, and once for a suppurating node on the parietal bone, a large portion of the outer table of which had exfoliated and been removed.

Examination of the chest detected chronic bronchitis, and on the right side, where he complained of pain, there was evidence of circumscribed effusion to a small extent, with dulness on percussion, and absence of respiration, not influenced by change of position. No enlargement of the liver was discovered, nor did sufficient evidence of organic disease present itself elsewhere, but the existence of Bright's disease of the kidneys was suspected. He had occasionally slight œdema of the face, about the eye-lids. Under treatment, the nodes disappeared, the diarrhœa ceased, the pain and effusion in the right side of the chest were removed, and his general aspect improved, but all the usual remedies failed in permanently checking the vomiting; creosote seemed to have some influence, but only temporary. It was very hard to point out the cause of this obstinate vomiting; there was no sign of disease of the brain; it had not the character of that attending scirrhus of the pyloris, nor was there pain or tenderness in the epigastrium. About a month after admission the lymphatic glands in the neck increased in size, and were painful. His voice, before of natural strength and tone, became weak and husky ; but it was not until he had been in hospital six weeks that he was discovered to have an aneurism of the carotid artery.

May 7th. He complained of having suffered from great throbbing in the glandular swelling in the left side of the neck. Beside the more superficial glandular enlargement with its fistulous openings, a deeper-seated tumour could be felt, soft, and containing fluid, but having a well-marked diastolic pulsation : it was partly on the inside of, and partly covered by, the sterno mastoid muscle. Pressure on it impeded respiration ; pressure on the carotid below it could not be borne, both on account of the pain and its inducing vomiting ; it could not, therefore, be tried long enough to empty the sac. There was no *bruit de soufflet.* The existence of the aneurism had not been observed before, probably on account of its having been, while small, masked by the suppurating glandular enlargement over it ; besides, he usually kept a poultice on the part, and, making no complaint, the whole attention was absorbed in the other more obtrusive and serious complaints. As the aneurism got larger, the glands got less, from both which causes its existence became more apparent. It was not painful or tender, but the pulsation distressed him, and the pressure on the side of the larynx produced cough of a wheezing, laryngeal character, and reduced the voice nearly to a whisper.

This man, with such a constitution, was obviously no subject for any operation with the knife ; in this my colleagues and myself fully agreed ; the cure by pressure was, in such a situation, of course, out of the question. Some months previously I had seen in one of the French journals an account of the application of galvanism and acupuncturation in curing an aneurism, by coagulating the blood in the sac. I then thought the plan sufficiently feasible to determine me to try it in the first eligible case.

The present case, cut off from the usual resources of art, was clearly one in which even a doubtful remedy might be fairly tried. I began to form the opinion, too, that, in the absence of any more obvious cause, the pressure of the aneurism on the par vagum might account for the obstinate vomiting.

May 15th. In the presence of my colleagues, Drs. Hutton, Macdonnell, and Macdowell, and Mr. Stapleton, of Jervis-street Hospital, I proceeded to apply the galvanism to the tumour. The state of the aneurism previous to the operation was as follows : it was about the size of a hen's egg, but rather flat, of somewhat irregular shape, with a round, smooth projection on the inside, where the walls of the sac appeared thinnest. The centre of the tumour was on a level with the cricoid cartilage, the sterno-mastoid muscle was stretched over it ; the pulsation was strong, but no *bruit de soufflet* was audible.

I passed a thin gold needle into the outside of the sac, till it had penetrated to about an inch; the same was then done on the inside, the thin projecting part of the aneurism being avoided; the needles could be made to touch in the centre. I used needles of gold, as better coagulators of blood than those of steel; by the advice of Mr. Fagan, the electrician to the hospital, who was kind enough to regulate the galvanic battery for me, they were isolated every where, except at the point, by a varnish of shell-lac. The battery used was one of Smee's, consisting of twelve zinc and silver plates. The action was given very gradually, by, at first, only immersing the plates to two or three inches. When the whole force of the battery was used, it only caused moderate pain, and produced slight contraction of the muscles; he compared the pain to the prick of a leech. At the end of fifteen minutes the coagulation of the blood was not such as to cause any remarkable change in the tumour, but it appeared to beat with less force at the outside. After this the pulsation became evidently less, the tumour firmer and larger, and he began to complain of uneasy, weighty sensations, and very severe pain, which he compared to his throat being held fast by the teeth of a dog. He said he suffered much from pain in the left side of the forehead, with lightness, and other unpleasant feelings. The sensations in the tumour were very distressing, and those in the head, from their violence, assumed rather an alarming character; but the most serious symptom was the great increase of the swelling as coagulation took place chiefly in a direction upwards and downwards, in the course of the sterno-mastoid muscle; this seemed the chief cause of the pain and the tight feel in the throat.

At the end of twenty-five minutes complete coagulation had taken place in the aneurism, which felt solid, and pulsation was imperceptible; for these reasons the galvanism was discontinued. The discontinuance might have been demanded also for another reason, that round the positive needle, on the outside, the parts in immediate contact were observed to vesicate, and then to turn quite black for the size of a spangle, the vitality being destroyed by the galvanic action: when this needle was withdrawn there was a slight flow of blood, but none from the puncture of the negative needle.

So far, therefore, as the solidifying of the blood in the aneurism, the operation had succeeded, but not without considerable grounds of uneasiness. The unpleasant feel in the head continued, with the pain over the left eye-brow; the pupil was observed to be contracted, and there was *loss of sight in the left eye.* He was restless, and tossed about, complaining much of the tightness of his throat; he had twitches in the lower extremities, and complained of being chilly, and the pulse fell from 74 to 60. With respect to the tumour itself, the sudden increase was sufficiently alarming, as it was three or four times larger at the termination of the application of the galvanism. The increase, as observed before, took place chiefly upwards and downwards in the course of the muscle; it reached from about one inch above the clavicle to an inch and a half below the ear. The tumour was also more prominent. From what did this increase arise?

May 16th. Has passed a sleepless night, and frequently vomited the iced brandy and water which he had been ordered. He had pain both in the head and in the tumour, and was unwilling to have the latter touched, it was so sore. Reaction had now set in, and the pulse was 86. The tumour was quite solid in every part, except at the inside, where it was softer, and where, I thought, I felt pulsation, but it was too indistinct to be certain. The whole solid body of the tumour was lifted up by each pulsation of the trunk of the carotid beneath it. He did not suffer pain in either the head or tumour; but complained of great weakness. Tongue rather dry, and thinly furred.

17th. Says he is better; no pain or throbbing sensation in the tumour; it looks large, I should say about four times larger than before the application of the galvanism. It feels hard and perfectly free from pulsation at its posterior half; but at its anterior half, where there is the sensation of fluid, pulsation is perceptible, but less strong than before. He does not now complain on its being touched. Where the positive needle was inserted, the small, round, black spot is observable; pulse 80; vomiting as before. After this the pulsation returned in the whole tumour, which, though much increased, resumed more of its original oval form, but became very prominent, the sterno-mastoid muscle being on the stretch across it.

A question now naturally presented itself,—should the application of the galvanism be repeated? My own impression decidedly was, that it should not, for should it be followed by a still further increase of size, in addition to its already large bulk, the pressure on the trachea and jugular vein might induce serious, if not fatal results. During a temporary absence in England, my colleague, Mr. Adams, who kindly took charge of the case, had a full consultation upon it, at which Sir P. Crampton was present, when it was decided that further interference by operation in such a constitution would only hurry on the fatal termination of the case.

The vomiting continued as violent as ever, and he died, apparently of exhaustion, on the 8th June, a little more than three weeks after the application of the galvanism. A few days before his death there was no pulsation in the aneurism.

*Post-mortem Examination.*—There was nothing found in the viscera to explain the vomiting, the stomach being only a little redder than natural, as was also a short portion of the ileum. The substance of the liver did not appear diseased, but it presented a curious malformation, being divided into a number of small lobules, like the kidney of an ox. No appearance of disease in the brain. The kidneys in the first stage of Bright's disease. The heart, aorta, and large vessels of the neck, were removed along with the aneurism, and carefully examined; the heart and aorta were healthy. The aneurism was about the size of a large orange, its superior surface was on a level with the upper edge of the hyoid bone, its lower with the seventh ring of the trachea; it was globular at its anterior two-thirds, flatter behind, between which two portions, on the outside, was a deep groove, partly filled by the edge of the sterno-mastoid, and partly by the jugular vein, which was quite flattened and impervious. The par vagum nerve ran over the posterior surface, at first expanding out into a fibrous arrangement, afterwards so flattened on the surface of the tumour that it formed a membrane a quarter of an inch broad, so thin and so closely applied, that it required delicate dissection to raise it off the wall of the aneurism, and trace it on to its trunk above the tumour, where it became normal.

The sac of the aneurism felt strong and rather thick, particularly in front, and as if its contents were in a great measure solid; posteriorly it had a softer and more fluid feel. It sprung from the anterior part of the common carotid artery, but the vessel was lost in the tumour, and only traceable a short distance up the lower part of the back. Below the aneurism, the trunk of the carotid was sound, but both external and internal carotids were much reduced in size, and so much obstructed that a probe could not be passed through them into the aneurism.

A section of the aneurism showed the contents to be solid, the centre occupied by clotted blood, of the colour and consistence of black currant jelly; from a quarter to half an inch from the margin the layers were of a pale red colour, and had a fibrous arrangement, exactly resembling muscle; when they were removed the lining membrane was found smooth but uneven.

As far as coagulating the blood in the sac, the application of the galvanism in this case was successful, complete coagulation having been effected by it. From the proximity of the carotid artery to the heart, and the direct course of its trunk (both favouring the rapid current of the blood,) as also from the very free anastomosis with the numerous branches of the corresponding artery, an aneurism in this situation is one least likely to preserve the coagulum formed by the galvanism. In the present instance, likewise, a successful result may have been prevented by the total impossibility of using sufficient pressure to obstruct the current, and prevent its washing away the newly-made clot. To be completely successful, repetition of the operation would have been required; my reasons for not deeming this advisable have been already stated. What I have observed, however, convinces me that in more suitable cases this mode of treating aneurisms will yet be found most valuable.* The sudden and rather alarming increase of the tumour, which occurred during the application of the galvanism, should it be constantly observed, may fairly be brought forward against its use in aneurism situated, as this one was, in the neighbourhood of important organs, which would be

* A case is given in the *Revue Medicale*, for December, 1842, of a popliteal aneurism in a man of seventy, cured by M. Petrequin, of the Hotel Dieu of Lyons, with acupuncture and galvanism, in a single sitting; and several cases have since appeared in the public journals.

very intolerant of sudden pressure, although they may bear or accommodate themselves (as we know they do) to the gradual pressure of tumours.

It is not easy to account satisfactorily for this rapid enlargement; the perfect integrity of the sac shows it was not from extravasation of blood by rupture; moreover, no traces of blood could be discovered. We know that during the galvanic action a quantity of hydrogen is evolved from the negative pole; it would, however, have been scarcely equal to the actual amount of the increase; the sensation, also, was of something more solid than if the contents were gaseous fluid. It now appears to me more likely to have been caused by the galvanic influence extending beyond the sac, and coagulating the fluids in the cellular tissue around it, the coagulated matter having been afterwards absorbed. The size of the aneurism at the time of death was certainly not larger than it would have been in the usual progress of the disease, and if the galvanism had never been applied.

M. Petrequin insists on the necessity of the needles crossing, to produce a proper coagulum. The needles, in this case, though they could be made to touch, certainly did not cross, and yet coagulation was complete. But I have further reasons for believing this is not necessary: I thought that, in performing the operation for the future, it would be as well to avoid, if possible, the entrance of the hydrogen gas evolved from the negative pole directly into the circulation. I therefore suggested to Mr. Fagan to make the experiment of putting an albuminous fluid into a small bladder, and to insert the positive needle into the fluid; but merely to apply the negative wire to the outside of the bladder. He accordingly filled a small portion of sheep's intestine with one part of white of egg and two parts water, quite full, and without any air. He inserted the positive needle its whole length through the gut into the fluid, and applied the negative wire merely to the outside of the sac, and succeeded in producing a large tea-spoonful of mucous-looking coagulum, without a bubble of hydrogen in the fluid inside, but many adhering to the outside, and to a silver plate on which the sac was placed. We have no grounds to say the entrance of hydrogen into the blood is injurious; but the fact that coagulation can be produced without its necessarily being present is interesting. The condition in which the par vagum was discovered may, perhaps, explain the incessant vomiting. It is scarcely possible to suppose that a nerve so closely connected with the functions of the stomach could be so much deranged in structure without considerable gastric disturbance.—*Dub. Quarterly Journal of Medical Science.*

